# Implementation and evaluation of a complex intervention to improve information availability at the interface between inpatient and outpatient care in older patients with multimorbidity and polypharmacy (HYPERION-TransCare) — study protocol for a pilot and feasibility cluster-randomized controlled trial in general practice in Germany

**DOI:** 10.1186/s40814-023-01375-2

**Published:** 2023-08-22

**Authors:** Astrid-Alexandra Klein, Jenny Petermann, Franziska Brosse, Steve Piller, Martin Kramer, Maria Hanf, Truc Sophia Dinh, Sylvia Schulz-Rothe, Jennifer Engler, Karola Mergenthal, Hanna M. Seidling, Sophia Klasing, Nina Timmesfeld, Marjan van den Akker, Karen Voigt

**Affiliations:** 1 Department of General Practice/Medical Clinic III, Dresden Medical School, Dresden University of Technology, Dresden, Germany; 2 Institute of General Practice, Goethe University, Theodor-Stern-Kai 7, 60590 Frankfurt Am Main, Germany; 3 Cooperation Unit Clinical Pharmacy, Department of Clinical Pharmacology & Pharmacoepidemiology, Heidelberg University Hospital, Im Neuenheimer Feld 410, 69120 Heidelberg, Germany; 4 Department of Medical Informatics, Biometry and Epidemiology, Ruhr University Bochum, Universitätsstraße 105, 44789 Bochum, Germany; 5 Department of Public Health and Primary Care, Academic Center for General Practice, KU Leuven, Leuven, Belgium; 6 Department of Family Medicine, School CAPHRI, Maastricht University, Maastricht, the Netherlands

**Keywords:** Polypharmacy, Multimorbidity, Family practice, Inpatient and outpatient care, Continuity of care, Participation, Older patients, Randomized controlled trial, Pilot projects, Information availability

## Abstract

**Background:**

Despite attempts to improve the cross-sectoral flow of information, difficulties remain in routine healthcare. The resulting negative impact on continuity of care is often associated with poor health outcomes, especially in older patients. Our intervention aims to increase information availability with respect to medications and health conditions at the interface between inpatient and outpatient care and to contribute towards improving the quality of care in older patients. This pilot study focuses on feasibility and implementability.

**Methods:**

The idea of the complex intervention has been developed in a previous study. This intervention will be tested in a prospective, multicenter, cluster-randomized (via web tool), controlled pilot trial with two parallel study arms (intervention and control group). The pilot study will be conducted in 20 general practices in Hesse and Saxony (Germany) and include 200 patients (≥ 65 years of age with multimorbidity and polypharmacy) recruited by the practices. Practice staff and patients will be blinded. We will use qualitative and quantitative methods to assess the feasibility and implementability of the intervention and the study design in a process evaluation covering topics ranging from expectations to experiences. In addition, the feasibility of proposed outcome parameters for the future definitive trial will be explored. The composite endpoint will include health-related patient outcomes (hospitalization, falls, and mortality using, e.g., the FIMA questionnaire), and we will assess information on medications (SIMS questionnaire), symptoms and side effects of the medication (pro-CTCAE questionnaire), and health literacy (HLQ questionnaire).

Data will be collected at study begin (baseline) and after 6 months. Furthermore, the study will include surveys and interviews with patients, general practitioners, and healthcare assistants.

**Discussion:**

The intervention was developed using a participatory approach involving stakeholders and patients. It aims to empower general practice teams as they provide patient-centered care and play a key role in the coordination and continuity of care. We aim to encourage patients to adopt an active role in their health care. Overall, we want to increase the availability of health-related information for patients and healthcare providers. The results of the pilot study will be used in the design and implementation of the future definitive trial.

**Trial registration:**

The study was registered in DRKS-German Clinical Trials Register: registration number DRKS00027649 (date: 19 January 2022).

Date and version identifier

10.07.2023; Version 1.3

**Supplementary Information:**

The online version contains supplementary material available at 10.1186/s40814-023-01375-2.

## Background

Information sharing at the interface between inpatient and outpatient care remains problematic [1–4]. Information can be incomplete, outdated, or not received at all. Communication and cooperation between healthcare providers are often described as insufficient, difficulties arise in contacting responsible healthcare personnel, and general practitioners (GPs) perceive a lack of appreciation [3, 4]. Deficiencies in informational continuity can be particularly problematic in older people, as increasing age is often accompanied by multimorbidity and polypharmacy. Moreover, age-related changes in medication tolerance, inappropriate polypharmacy (overuse, underuse, and misuse of medications), and poor adherence increase the risk of potentially preventable harms (e.g., adverse drug reactions) and rising healthcare costs [5–7]. In addition to more visits to the doctor, this is also associated with more frequent and longer hospital admissions [1, 8], leading to unintentional interruptions in necessary therapies and to unexplained changes in drug therapies [1]. At the interface between inpatient and outpatient care, this may result in major problems such as medical errors, inappropriate treatment, patient anxiety, and a lack of trust in healthcare [4, 9]. At the same time, older patients with multimorbidity, polypharmacy, and complex care needs are already vulnerable and often have poorer outcomes, such as longer and more frequent hospital stays, a higher risk of adverse drug events and falling, lower quality of life, and higher mortality rates [8, 10, 11].

They also experience poorer healthcare compared to patients without multimorbidity due to the complexity of their conditions and the overall care process. This complexity also makes it difficult to develop evidence-based guidelines [8, 12].

International studies have also shown that medication discrepancies and discontinuations, and adverse events, frequently occur at the interface between inpatient and outpatient care. Many of these adverse events are preventable and can have negative consequences for patients such as discomfort, clinical deterioration, rehospitalization, and even death [13–15].

Since GPs generally take a more holistic view of patients [16, 17], provide patient-centered care, and play a key role in the coordination and continuity of healthcare, they are particularly well-positioned to treat patients with multimorbidity and polypharmacy and coordinate their care. About 80% of GPs’ consultations are with this patient group [16].

Results from two pivotal German trials have been considered in the development of this study protocol: the Frankfurt cluster-randomized controlled trial PRIMUM (PRIoritization of MUltimedication in Multimorbidity) [18–20], which tested a complex intervention in GP practices, and the VESPEERA project [20, 21], which tested a complex intervention at the transition of care between GP practice and hospital. The studies failed to prove the effectiveness of the interventions because of methodological challenges, including coordination difficulties between healthcare providers, and because patients’ baseline functional capacity was high (ceiling effect) [18–20]. Furthermore, levels of implementation in the GP practices were low [21]. In the literature, on the other hand, existing deficits in care and the positive effects of patient empowerment have been demonstrated [21, 22], and it has been shown that improving continuity of care can lead to better health processes and outcomes [22, 23].

Patients’ role in sharing their personal health-related information at the interface between inpatient and outpatient care could be improved in the German healthcare system by increasing their involvement and empowerment, both of which patients would welcome [21]. Schaeffer et al. [24] showed that health literacy in Germany is generally low, especially among older people and people with multimorbidity. Moreover, digital health literacy is particularly low, especially among older people and people with a low level of education and/or social status. Even during the COVID-19 pandemic, older people showed no overall improvement in digital health literacy, unlike younger people. Furthermore, the free choice of practitioner in the German healthcare system increases the complexity. Patients are free to consult any healthcare service or healthcare provider, like GPs and other medical specialists without having to share any information about prescribed and received services with providers [25]. In Germany, relaying personal health information is limited due to strict data protection requirements [25], and so far, the implementation of IT solutions, e.g., an electronic patient record (ePA in Germany), is lagging behind [26]. As a result, patients are often the messenger of healthcare information between different providers [27].

The design of the HYPERION-TransCare project was based on the findings and experiences described above. The target group consists of older people with multimorbidity and polypharmacy.

In our first qualitative study, the challenges and difficulties, but also ideas for solutions and improvements, at the interface between inpatient and outpatient care were identified and discussed with patients and major stakeholders. A participatory approach was used in developing and coordinating an intervention and supporting materials with representatives from outpatient care (GPs, healthcare assistants (HCAs), outpatient nurses), inpatient care (clinical doctors, clinical nurses, hospital pharmacists, clinical information scientists), patients, informal caregivers, and patient representatives [28].

The overall result was a complex intervention that aims to empower and encourage patients to collect and share their own data with healthcare professionals and to strengthen the role of GP practices in coordinating care. To further improve the transition from outpatient to inpatient care, the intervention also aims to optimize the information availability on medications and health conditions between the different care settings. We expect that the future definitive trial will provide indications of the functioning of these processes already described above.

As this is a participatory, newly developed complex intervention, we expect it to be more widely accepted and easier to implement. Nevertheless, it is important to test it first in a pilot study to gain insight into acceptance, implementability, and suitability and to make adjustments if necessary, thus contributing to the success of the future definitive trial [18, 29]. That is why we designed this pilot trial and present the study protocol here.

### Objectives and hypotheses

This pilot study aims to test the feasibility and implementability in a sample of patients and their GPs in an exploratory mixed-methods process evaluation of (a) the intervention and (b) the study design and outcome measurements. Additionally, it aims to provide preliminary results on effectiveness of the intervention.

## Methods/design

An exploratory, prospective, cluster-randomized pilot study (including process evaluation) will be conducted. The multicenter two-arm study (parallel groups) will be conducted in 20 GP practices in Hesse and Saxony (Germany), whereby each practice will be asked to recruit 10 patients, bringing the total to 200 patients.

We will compare the results of the complex intervention for improving information availability in preparation for the transition of care in an intervention group (IG), with the results of a control group (CG) that will receive usual care, i.e., in accordance with German GP practice guidelines [30, 31].

The study complies with the Declaration of Helsinki and was approved by the Ethics Committee of the Technical University of Dresden, the Ethics Committee of the Department of Medicine of Goethe University Frankfurt am Main, and the Ethics Committee of the Medical Faculty of the University of Heidelberg. The study protocol follows the SPIRIT 2013 guidelines [32], adapted according to Thabane und Lancaster (2019) [33] (see Additional file 1) and the CONSORT 2010 statement [34] (see Additional file 2) and was registered in the German Clinical Trials Register (DRKS).

### Recruitment/inclusion and exclusion criteria for participants

Practices will be recruited primarily through SaxoForN [35, 36], which is a German practice-based research network (PBRN [37]) that is being established in Saxony and Hesse and GP practices associated with the study teams’ institutes. Participation is open to general and internal medicine practices that provide full care (incl. home visits) and did not participate in our first qualitative study.

GP practice teams (GP and HCA) will receive an online educational training followed by a telephone call (training 1) to prepare them for patient recruitment. The GP practice teams will then use a standardized procedure to recruit patients over a period of 3–4 weeks. The inclusion criteria are as follows: aged ≥ 65 years, two or more chronic diseases, five or more chronic medications, ≥ one hospitalization in the past 12 months, and sufficient knowledge of German (reading and language comprehension). Patients that are unable to provide consent, live in a nursing home, suffer from diagnosed severe mental disorders (ICD-10 F diagnoses), or that participated in our first qualitative study will be excluded.

Each GP practice will filter as many of these criteria as possible using their practice management system and will create a list of patients that fulfill them. The filtered criteria and number of patients will be documented. Letters will then be sent to patients by post to invite them to participate. Only patients that have a regular appointment in the practice within the specified period of 4 weeks will be invited. In view of the future definitive trial, two slightly different approaches will be used in Hesse and Saxony with the aim to compare and to examine their feasibility. In Hesse, patients fulfilling the study criteria will be invited in the order of their appointments in the practice, starting with the first 20 patients. In Saxony, after filtering the criteria, the study team will then assign an individual random letter taken from the alphabet to the practice. The GP practice will check the criteria that have not been filtered yet, starting with the first patient whose name begins with that letter. This process will be repeated for every fifth patient on the entire list until 20 patients have been identified.

If fewer than 10 patients show interest/agree to participate in the study, additional patients will be contacted from the list according to the process described above until the targeted 10 patients per GP practice is reached, or the study recruitment period has ended. Patients will be included in the study after receiving comprehensive study information from their GP and once they have signed the informed consent form. If necessary, friends or family members can help patients to participate in the study. An assistant from the study team will support the recruiting process, and a study hotline will be set up.

### Allocation and blinding

To avoid contamination of the IG and CG, the level of randomization will be the GP practice (cluster randomization) stratified based on study site. Upon inclusion, each GP practice will be allocated a consecutive number by the study team (pseudonym). In preparation for block randomization, an online tool [38] will be used to generate a random list of the two study arms in blocks of 6 and 4 for each study region. The pseudonym of the practices will be entered into the randomization list in the order in which participating patients from a specific GP practice complete the baseline surveys (t0). The GP practice will thus be randomly assigned to one of the study arms. The randomization process will be administered confidentially by two researchers from one of the study teams’ institutes that are not involved in the study.

All participants will be blinded. After block randomization, GP practices will receive study materials and training according to their assigned study arm (training part 2, digital). After completion and evaluation of the study, all participants will receive a booklet containing an overview of the methodology and main results of the study. The statistical analysis will be performed by blinded staff at Ruhr University Bochum (cooperation partner).

### Intervention/study arms

The 6-month intervention period for each GP practice will begin after practice randomization has been completed. The study will be conducted under everyday conditions in routine care. Accordingly, there is no concomitant care permitted or prohibited during the trial.

In both study arms, both GPs and HCAs will be free to decide individually who completes what study task.

#### Intervention group

**Fig. 1:**
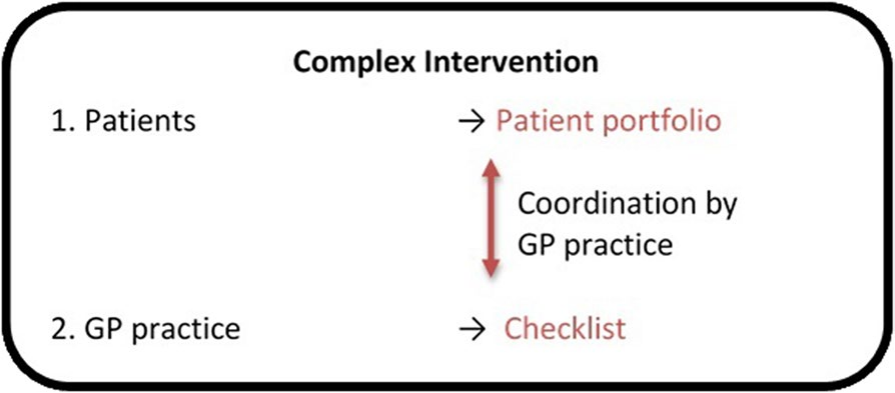
The complex intervention in HYPERION-TransCare

The planned complex intervention is shown in Fig. 1. It consists of several components that are described in more detail below.

### Patients

Each patient in the IG will receive a “patient portfolio.” The aim of the portfolio is to help patients collect and organize important, up-to-date, personal, medical, and health-related data so that both, they and their healthcare providers, are well informed. The content of the patient portfolio matches the “GP practice Checklist” (see below) and will include a sheet with “Instructions for Use” as well as sleeves and dividers for organizing the documents. A sheet containing “key patient data” will summarize important health information for a brief overview of what is contained in more detail in the portfolio.

Furthermore, the portfolio will include a patient flyer developed by staff at the Heidelberg University Hospital (cooperation partner) that contains general advice on sharing information with healthcare providers. The flyer was based on various established patient brochures [39–41].

In a first step, patients will complete the patient portfolio themselves. However, the GP practices will provide patients with support if necessary. They will be instructed to take their portfolio to their healthcare providers and to keep it updated. The healthcare providers thus have access to new information (e.g., medical findings, reports) or can add information to the portfolio themselves. GPs and HCAs will be asked to assist patients in maintaining and managing their patient portfolios at routine appointments. This intervention should strengthen patients’ self-management skills and improve knowledge about their personal health status. The structured collection of relevant documents and information and in particular the take along should improve the availability of this information for healthcare providers.

The patient portfolio and the patient flyer were reviewed and revised by “Was hab' ich?” gGmbH (who is specialized in translating medical information into plain language) to ensure comprehensibility for patients. Furthermore, three patients, two GPs, and two HCAs evaluated the portfolio with regard to completeness, manageability, and comprehensibility in telephone interviews. The portfolio was then adapted and finalized accordingly.

### GP practices

For the implementation of the complex intervention, GP practices in the intervention group will receive a “GP practice Checklist,” which will be used separately for each study patient. The checklist can be completed and saved on paper or digitally, depending on preference. The checklist helps the GP practice administer and complete the *patient portfolio* as well as to complete the information in the GP practice´s documentation. It contains a list of information (e.g., disease-specific data, information on medication, personal data, documents, special features, and notes) that is important for both the GP practice and patients as well as in case of hospitalization. The date on which the checklist and notes were last updated will also be entered. The checklist includes an indication whether the information is more likely to be provided by the GP practice or the patient.

In a pretest, the checklist was evaluated in a telephone interview with two GPs and two HCAs with regard to completeness, manageability, and layout and was then adapted and finalized. The intervention aims to support the patients’ self-management skills and knowledge about their health status. It should furthermore contribute to a more complete set of information on the side of the patient and in turn more comprehensive information for healthcare providers.

In addition, the GP practice will receive information from the abridged version of the German GP guidelines on polypharmacy and multimorbidity [30, 31].

#### Control group

During the 6-month intervention phase, treatment for participants (GP practices and patients) randomly assigned to the CG will be “care as usual.” This means that treatment will be provided in accordance with established evidence-based medical guidelines. As in the intervention group, medical guidelines for the management of multimorbidity and polypharmacy (for GP practices), as well as the patient flyer (for patients), will be provided to the control group. This procedure will serve to blind the participants.

### Adherence

In order to increase adherence among participants, the study team will provide the GP practice with close methodological support in the shape of educational trainings; regular communication via telephone, e-mail, and practice visits if necessary; and additional support during further stages of the study (study hotline). The study team will also regularly monitor digital data entered onto the research platform REDCap (see below). Patients in the intervention and the control groups will both be supported by the GP practice and by the study team through contact as well as interviews. Furthermore, all participants will receive compensation when they have successfully completed the baseline survey (patients 50 €; GPs and HCAs will each receive 50 € per study patient). This time point was chosen to encourage participants to complete the study.

### Benefits and harms of the intervention

As a result of study participation, patients in the IG are expected to learn more about their health and treatments, which should help them in the personal management of their diseases, while GP practices are expected to improve scope and quality of information on patients, which should enable them to improve care. Since the study involves no medical interventions, no direct medical risks are associated with participation.

For participants in the IG, risk may be associated with the health-related information if, for example, it is incorrect or incomplete. When planning the study, we attempted to avoid these risks by, for example, coordinating the monitoring of study participants by GP practices and the involvement of the study team in providing study assistance. Moreover, these risks, while independent of the study, may also occur in routine care. Overall, the benefits are expected to outweigh the risks. No additional risk will be associated with participation in the CG, as participants will continue to receive care as usual.

### Explorative process evaluation of piloting the implementation of the complex intervention: data collection methods and feasibility outcomes

As described above, the pilot study consists of the implementation of the developed intervention components and study design, as well as its accompanying exploratory process evaluation. As this is a pilot study, the process evaluation will contribute to better understand how distinct intervention components were implemented and whether the implementation followed the protocol. The process evaluation was conceptualized in collaboration with the Department of Clinical Pharmacology and Pharmacoepidemiology, Heidelberg University Hospital. The explorative mixed-methods approach will follow the framework for designing and reporting on process evaluations of cluster-randomized trials (Grant et al. [42]) and the Consolidated Framework of Implementation Research (CFIR, Damschroder et al. [43]).

Its design consists of an evaluation of routinely collected study data (e.g., how many GPs were contacted/how many were recruited/how many patients completed the intervention; what were common reasons for nonparticipation), maintenance and use of intervention components, and outcome measures (e.g., use and application of checklists and patient portfolios, as well as adherence to fulfill questionnaires, plausibility checks, and missing analyses). Additionally semi-structured interviews about the participant´s comprehensions, expectations and experiences regarding barriers and facilitators, e.g. what worked well and what were problems during each phase of the study, will be performed (see Additional file 3).

The semi-structured interviews will be jointly performed by trained interviewers of the Heidelberg team and the study team. GP practice staff and the patients from both intervention and control group are interviewed in total at five timepoints. Interview guides differ slightly in content depending on the study arm (IG or CG). Therefore, question domains and formulation suggestions were selected from the frameworks, translated into German, and adapted to the given settings in consensus with the study team.

### Feasibility of the outcomes measuring for the future definitive trial

In the pilot trial, we are testing the *primary endpoint* for the future definitive trial, which will be the combined endpoint of health-related patient outcomes (hospitalization, falls, and mortality), as shown in Table 1. *Secondary endpoints* for the future definitive trial will include improvement in information on medications (SIMS-D questionnaire [44]), a reduction in symptoms and/or side effects of the medication (pro-CTCAE questionnaire [45, 46] adjusted), an improvement in health literacy (HLQ-G questionnaire [47]), and improving the medication and treatment-related information: comparing the available information regarding the care situation between GP and patient (FIMA [48, 49]).

**Table 1:** Description of the combined endpoint for the future definitive trial

**Endpoint (quantity)**	**Patients**		**GP Practice**	
	**Data**	**Instrument**	**Data**	**Instrument**
Hospitalization	X	Single question regarding hospitalization including frequency and length of stay from FIMA [48, 49]	X	Single question regarding hospitalization including frequency and length of stay from FIMA [48, 49]
Falls	X	Standardized documentation (self-report)		
Mortality			X	Standardized documentation

### Data collection methods for the planned outcome measures for the future definitive trial

**Fig. 2:**
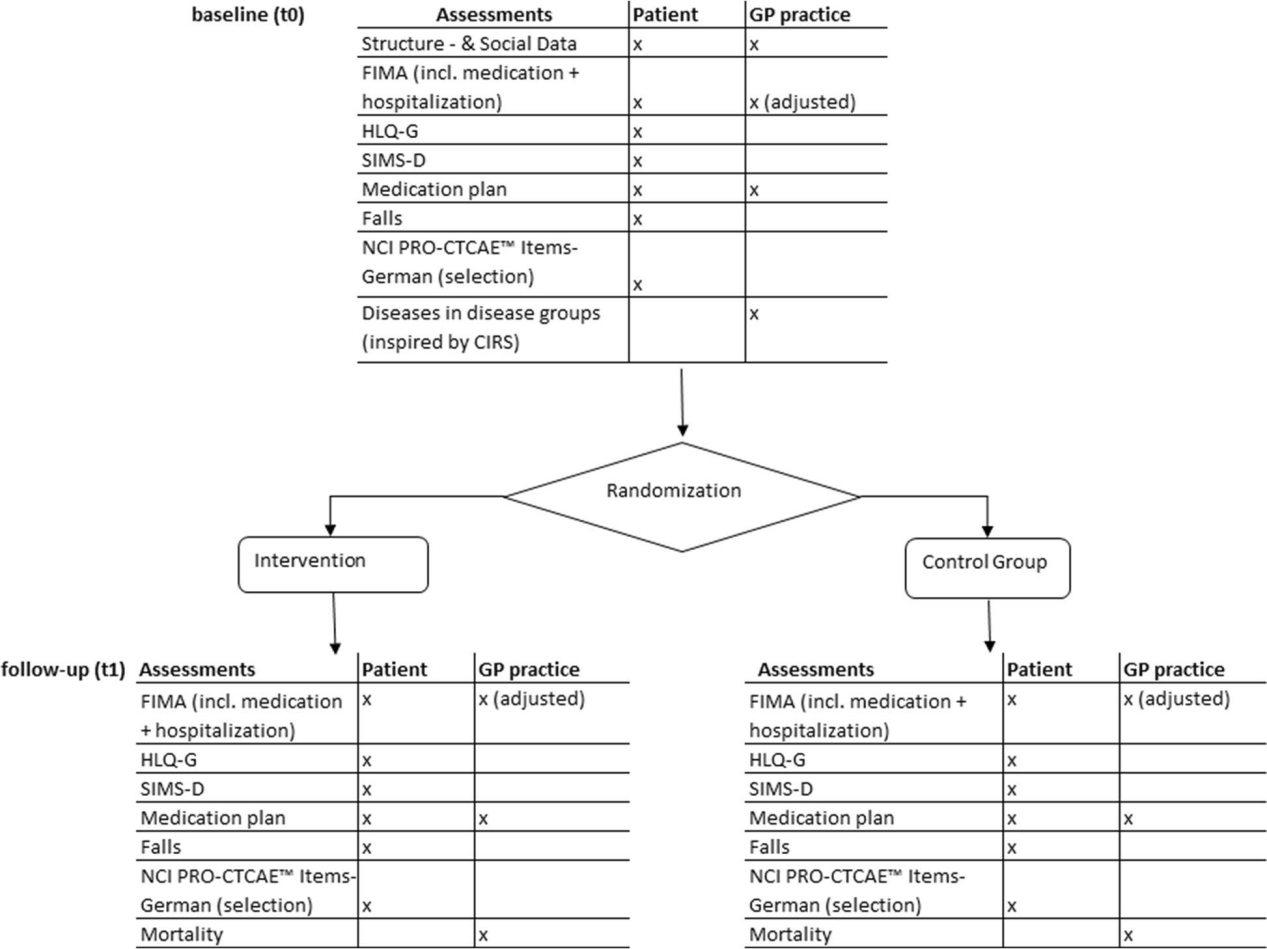
Assessments conducted as part of HYPERION-TransCare

In addition to the process evaluation, an overview of what data will be collected and when is shown in Fig. 2. Data are collected for both the IG and the CG.

#### GP practice

At *baseline*, information on the practice (such as practice size, practice type, and location), as well as sociodemographic information for the GPs and HCAs (such as sex, age, professional experience), will be collected using a self-administered questionnaire. Furthermore, the diseases of each included patient will be collected by 14 disease groups (based on the “Cumulative Illness Rating Scale” (CIRS) [50]).

At both *baseline* and *follow-up*, the standardized and validated FIMA questionnaire [48, 49] will be collected for each included patient. The FIMA (German acronym for “Questionnaire for Health-Related Resource Use in an Elderly Population” — Fragebogen zur Erhebung von Gesundheitsleistungen im Alter) “collects the health-related resource use within the older population groups” [48]. The questionnaire will be slightly modified, so that the questions are suitable for use in GP practices and reflect the patient data that is available to them. As an alternative to entering medications in the FIMA, practices may also send a copy of an existing medication plan to the study team. Mortality will be surveyed during the study (standardized documentation).

#### Patients

At *baseline*, a self-administered questionnaire will be used to collect data on sociodemographic characteristics (such as sex, age, and living situation), as well as knowledge and use of an electronic patient file (ePA in Germany). At both *baseline* (t0) and *follow-up* (t1), a medication plan will be required, along with information on the number of falls within the last 3 months (based on the WHO definition [51]) and on symptoms and/or side effects of the medication (selection of the German patient-reported outcomes version of the Common Terminology Criteria for Adverse Events (NCI PRO-CTCAE™ Items-GERMAN [45, 46]). Furthermore, validated and standardized questionnaires will be used:

- FIMA: Fragebogen zur Inanspruchnahme medizinischer und nicht-medizinischer Versorgungsleistungen im Alter (Questionnaire for Health-Related Resource Use in an Elderly Population) [48, 49]
- HLQ-G: Health Literacy Questionnaire — German [47]
- SIMS-D: Satisfaction with Information about Medicines Scale, German version [44]

Patient enrolment is scheduled to start in March and will be completed in May 2022. Data collection is expected to end in November 2022. The participant timeline is shown in Table 2.

**Table 2:**
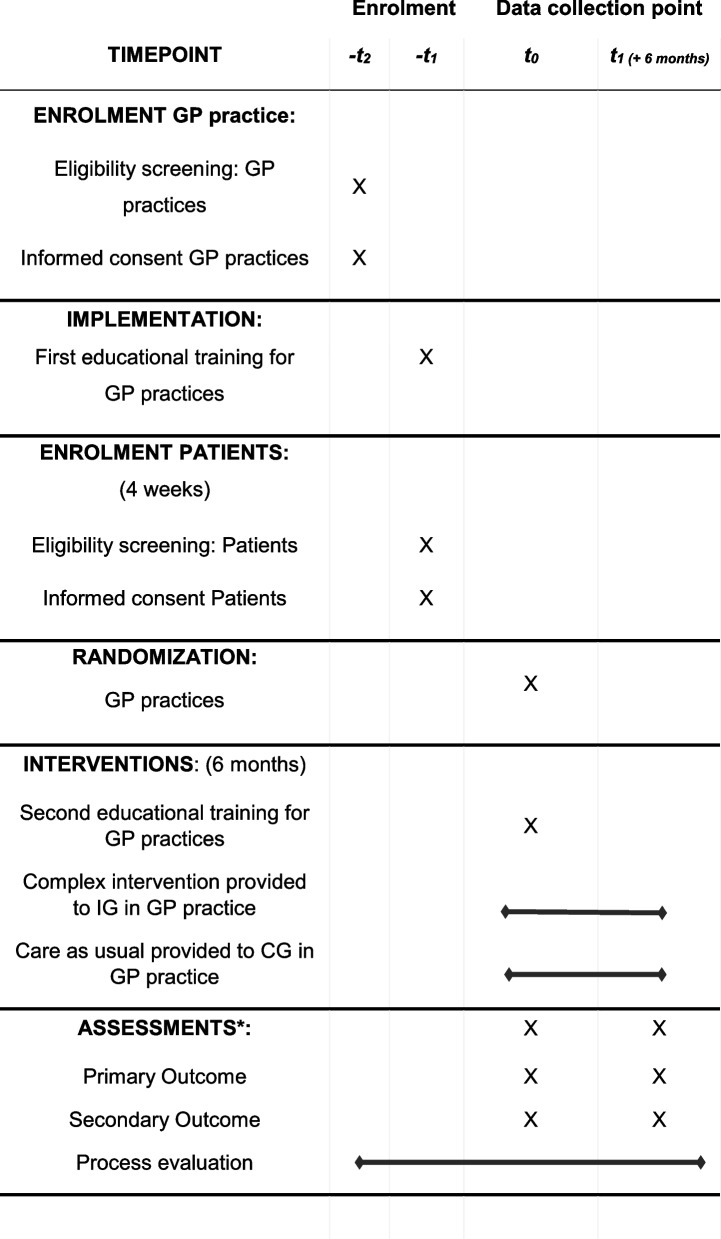
Participant timeline (SPIRIT 2013 diagram [32])

^*^For exact list of assessments, see Fig. 2

### Sample size

Since this is a pilot study, we did not perform a sample size calculation [29]. A number of 10 practices per site (Saxony and Hesse) as well as 10 patients per practice seemed to be a practical value that would be sufficiently informative regarding the feasibility of the study, even with some dropouts.

### Data management

Surveys conducted in the GP practices will take place digitally using an Electronic Data Capture-System (REDCap; hosted via the Coordination Center for Clinical Studies of the TU Dresden (KKS); storage on German servers). Patients will receive paper-based questionnaires (baseline questionnaire issued by GP practices; follow-up questionnaire sent by the study team), which will be returned directly to the study team in stamped addressed envelopes. The study team will then enter the data into REDCap. Semi-structured interviews with patients and GP practices will be conducted by telephone, recorded, and then transcribed verbatim.

A data protection concept was developed to take into account the legal directives (EU General Data Protection Regulation, national data protection law (Federal Data Protection Act) as well as regional directives, i.e., the Hessian and Saxonian data protection laws). In addition, an agreement on the joint processing of personal data was concluded between the study teams. The data will be treated confidentially at all times, and pseudonymized data will not be provided or made available to third parties.

According to the FAIR principles [52], anonymized data sets can be provided to others for future research. The data will only be passed on if certain rules are observed (e.g., approval by an independent ethics committee, use in medical research, application for use). The management of the two study teams will carefully review applications and decide, according to standardized criteria, whether to provide the data to the interested research group for the described research purpose.

Study consent can be withdrawn by practices or patients at any time without giving reasons to the study team and at no disadvantage to themselves. In this case, no further data will be collected, and existing data will be deleted. However, consent withdrawal will not affect data that has already been analyzed. After termination or discontinuation of the study, the data will be archived for up to 10 years in the protected databases of the two study teams. The databases will be secured against unauthorized access.

Participants are monitored regularly (e.g., telephone calls, checked REDCap entries). Irregularities noticed in the course of the study will be systematically documented or recorded as part of the process evaluation. Nonparticipants (date, sex, year of birth, reasons) as well as withdrawals and study discontinuations (date and reason, if known) will be documented. A dropout is defined as a patient that changes GP practices during the study phase, is admitted to a nursing home, or is no longer interested in participating in the study. Follow-up recruitment to compensate for dropouts will not take place because of the pilot study design. The analysis of the participation processes should provide important information to help in the design of the subsequent future definitive trial.

A plausibility check will be performed via REDCap. Data checks will ensure the data is of high quality. Data cleaning will be carried out independently of the study team by the cooperation partner of Ruhr University Bochum.

### Analysis

Wherever appropriate, interview transcripts will undergo content analysis based on Mayring [53] and through the use of MAXQDA. Quantitative and qualitative data will be analyzed descriptively with focus on the following:

1. *Feasibility*
  - The recruitment and randomization process will be presented descriptively. Adherence to the planned way of realization will be assessed. Furthermore, a descriptive analysis will be performed to compare the two study groups.
  - Content-oriented description of participants’ comprehension of and adherence to the intended implementation of the intervention’s components, their expectations, and experiences throughout the study period
  - Analysis for the maintenance of blinding will be done at patient and cluster level. At a patient level, mixed logistic regression analysis will be performed with dependent variable “patient correctly guess treatment group” and independent variable treatment group and a random cluster effect. Similar, at cluster level, logistic regression analysis will be performed with outcome “GP correctly guess treatment group” and independent variable treatment group. If the random cluster effect in the analysis at patient level is large, we will further explore the association between GPs guess and patients guess through logistic regression.
2. *Exploration of possible effects*: One possible effect of the intervention is an improvement in health literacy, as assessed using the HLQ-G. A linear mixed model that employs intervention group, HLQ-G at baseline, age, sex, and cluster as the independent variables will be used to evaluate the effects.
3. *Analysis of the combined endpoint*: A mixed logistic regression model will be used to analyze the planned combined endpoint, which is a combination of death, hospitalization, and falls (dichotomous), similar to the analysis planned in the main trial. In addition to study group, age and sex will be included as fixed effects, with the cluster added as a random effect. If a significant proportion of patients (> 10%) experience multiple events for falls and hospitalizations, we will perform additional separate analyses for these outcomes that uses a mixed negative binomial model. The sample size calculation for the future definitive trial will be based on the results of these models. Sensitivity analyses will be performed for per-protocol group.
4. Agreement and differences in FIMA results between patients and GPs will be analyzed using agreement parameters such as Kappa statistics.

The resulting assessment of the clinical relevance of the outcomes and the findings of the process evaluation will be used in planning the future definitive trial. Statistical analyses of intervention effects will be performed as part of the future definitive trial. Exploratory analyses will be performed according to the intention to treat principle.

## Discussion

Our study will address the need to ensure high-quality information is available at the interface between inpatient and outpatient care and available to the patients themselves. Literature shows that insufficient information is associated with preventable adverse health events [1, 4, 8, 9, 13–15]. Our intervention therefore aims to empower patients and GP practices by improving information on patients’ health conditions. Furthermore, the study results will provide valuable feedback to further develop the German electronic patient record ePA: focusing (a) which health-related data are the most important, (b) who could provide respectively which sources could be used for this, (c) aspects of data economy, and (d) user acceptance.

Other studies have already shown the importance of piloting large-scale intervention studies in advance [18]. By using a pilot and feasibility cluster-randomized controlled trial, we will gain insights and gather experiences that will help plan and implement the future definitive trial. This will include information on the actual use of the intervention documents and feedback from the GP practice teams on whether parts of the intervention (e.g., “GP practice Checklist”) should be digitalized further in the future definitive trial. Furthermore, we will gain insight in feasibility and on effectiveness of the outcome measures.

Even if the intervention can be integrated well into daily routines, it may be time-consuming. However, we assume that the intervention will improve informational continuity of care and possibly simplify the existing coordination tasks of GP practices. In the long term, this should save time and speed up the exchange of information with other healthcare providers and patients.

Results will be disseminated via publication in open-access peer-reviewed scientific journals and at congresses and conferences. Furthermore, a project report on the results will be presented to the funder, and a booklet containing an overview of the study will be developed in plain language for participants and the general public. There are no restrictions on publication.

### Strengths and limitations

The intervention was developed using a participatory approach, i.e., with the involvement of stakeholders that are directly affected by or confronted with the challenges and impacts of the availability of health information in everyday life and routine care [28]. The intervention should therefore be easy to integrate into daily practice routines and procedures. The relevant participants/addressees in this pilot study will consist of the same stakeholder groups (healthcare providers and patients). In GP practices, study tasks will be shared among teams of GPs and HCAs, with both professions involved and responsible for carrying out important tasks. The results will show exactly how these tasks are divided up within the teams and whether and how the implementation can be integrated into daily practice routines.

Participating GP practices will receive study information step-by-step to minimize selection bias in patient recruitment. The participants and data analysts will all be blinded.

The study may be negatively affected by difficulties recruiting participants as a result of the COVID-19-related burden on GP practices of the vaccination campaign, discussions about compulsory vaccinations, potential practice closures, increased testing volume (PCR tests — polymerase chain reaction test) and sick leave among personal. However, the timetable set by the funder cannot be postponed any further, especially as it is not possible to predict when and how the situation may change in the future.

## Conclusion

The present study addresses an important topic in everyday care and aims to test the feasibility of a complex intervention aimed at improving information continuity at the interface between inpatient and outpatient care with the participation of patients. It will further investigate the feasibility of the study design. The results will be used to plan the future definitive trial, which will examine both the efficacy and cost-effectiveness of the intervention.

### Supplementary Information

**Additional file 1. **SPIRIT 2013 guidelines.

**Additional file 2. **CONSORT 2010 statement.

**Additional file 3. **Mixed-Methods approach of explorative process evaluation.

## Data Availability

Materials (in German language) and data (fully anonymized) generated in this study are available from the corresponding author upon reasonable request.

## Abbreviations

CG: Control group
GP: General practitioner
HCA: Healthcare assistant (German job title, includes 3 years of professional training). HCAs perform a variety of tasks (organizational, supportive, link between doctors and patients)
IG: Intervention group
PBRN: Practice-based research network
t0: Baseline surveys
t1: Follow-up surveys
