# Supplementary material for: Implementation and evaluation of a complex intervention to improve information availability at the interface between inpatient and outpatient care in older patients with multimorbidity and polypharmacy (HYPERION-TransCare) — study protocol for a pilot and feasibility cluster-randomized controlled trial in general practice in Germany
Source: Pilot Feasibility Stud. 2023 Aug 22;9:146. doi: 10.1186/s40814-023-01375-2 (PMC10463488; doi:10.1186/s40814-023-01375-2)
Supplement: Supplementary file 2 — Additional file 2. CONSORT 2010 statement. [file 40814_2023_1375_MOESM2_ESM.docx]

**
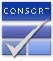
**

**CONSORT 2010 checklist of information to include when reporting a pilot or feasibility randomized trial in a journal or conference abstract**

| **Item** | **Description** | **Reported on line number** |
| --- | --- | --- |
| Title | Identification of study as randomised pilot or feasibility trial | ✓ |
| Authors * | Contact details for the corresponding author | ✓ |
| Trial design | Description of pilot trial design (eg, parallel, cluster) | ✓ |
| Methods |  |  |
| Participants | Eligibility criteria for participants and the settings where the pilot trial was conducted | ✓ |
| Interventions | Interventions intended for each group | ✓ |
| Objective | Specific objectives of the pilot trial | ✓ |
| Outcome | Prespecified assessment or measurement to address the pilot trial objectives** | ✓ |
| Randomization | How participants were allocated to interventions | ✓ |
| Blinding (masking) | Whether or not participants, care givers, and those assessing the outcomes were blinded to group assignment | ✓ |
| Results |  | Not appl. |
| Numbers randomized | Number of participants screened and randomised to each group for the pilot trial objectives** |  |
| Recruitment | Trial status† |  |
| Numbers analysed | Number of participants analysed in each group for the pilot objectives** |  |
| Outcome | Results for the pilot objectives, including any expressions of uncertainty** |  |
| Harms | Important adverse events or side effects |  |
| Conclusions | General interpretation of the results of pilot trial and their implications for the future definitive trial | ✓ |
| Trial registration | Registration number for pilot trial and name of trial register | ✓ |
| Funding | Source of funding for pilot trial | In the declarations at the end |

Citation: Eldridge SM, Chan CL, Campbell MJ, Bond CM, Hopewell S, Thabane L, et al. CONSORT 2010 statement: extension to randomised pilot and feasibility trials. BMJ. 2016;355.

**this item is specific to conference abstracts*

***Space permitting, list all pilot trial objectives and give the results for each. Otherwise, report those that are a priori agreed as the most important to the decision to proceed with the future*

*definitive RCT.*

*†For conference abstracts.*


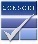
CONSORT 2010 checklist of information to include when reporting a pilot or feasibility trial*

| Section/Topic | Item No | Checklist item | Reported on page No |
| --- | --- | --- | --- |
| Title and abstract | | | |
|  | 1a | Identification as a pilot or feasibility randomised trial in the title | ✓ p. 1 (Title) |
|  | 1b | Structured summary of pilot trial design, methods, results, and conclusions (for specific guidance see CONSORT abstract extension for pilot trials) | ✓ p. 6 (Abstract) |
| Introduction | | | |
| Background and objectives | 2a | Scientific background and explanation of rationale for future definitive trial, and reasons for randomised pilot trial | ✓ p. 7 (Background) |
|  | 2b | Specific objectives or research questions for pilot trial | ✓ p. 10 (Background/Objectives and Hypotheses) |
| Methods | | | |
| Trial design | 3a | Description of pilot trial design (such as parallel, factorial) including allocation ratio | ✓ p. 10 (Methods/Design) / p.12 Allocation and Blinding) |
|  | 3b | Important changes to methods after pilot trial commencement (such as eligibility criteria), with reasons | not appl. |
| Participants | 4a | Eligibility criteria for participants | ✓ p.11 (Recruitment / Inclusion and Exclusion criteria for participants) |
|  | 4b | Settings and locations where the data were collected | ✓ p. 10 (Methods/Design) |
|  | 4c | How participants were identified and consented | ✓ p. 11 (Recruitment / Inclusion and Exclusion criteria for participants) |
| Interventions | 5 | The interventions for each group with sufficient details to allow replication, including how and when they were actually administered | ✓ p. 13 (Intervention / Study arms) |
| Outcomes | 6a | Completely defined prespecified assessments or measurements to address each pilot trial objective specified in 2b, including how and when they were assessed | ✓ p. 17 (data collection methods and feasibility outcomes) / p. 19 (Data collection methods for the planned outcome measures) |
|  | 6b | Any changes to pilot trial assessments or measurements after the pilot trial commenced, with reasons | not appl. |
|  | 6c | If applicable, prespecified criteria used to judge whether, or how, to proceed with future definitive trial | not appl. |
| Sample size | 7a | Rationale for numbers in the pilot trial | ✓ p. 22 (Sample size) |
|  | 7b | When applicable, explanation of any interim analyses and stopping guidelines | not appl. |
| Randomisation: |  |  |  |
| Sequence  generation | 8a | Method used to generate the random allocation sequence | ✓ p.12 (Allocation and Blinding) |
|  | 8b | Type of randomisation(s); details of any restriction (such as blocking and block size) | ✓ p.12 (Allocation and Blinding) |
| Allocation  concealment  mechanism | 9 | Mechanism used to implement the random allocation sequence (such as sequentially numbered containers), describing any steps taken to conceal the sequence until interventions were assigned | ✓ p.12 (Allocation and Blinding) |
| Implementation | 10 | Who generated the random allocation sequence, who enrolled participants, and who assigned participants to interventions | ✓ p.12 (Allocation and Blinding) |
| Blinding | 11a | If done, who was blinded after assignment to interventions (for example, participants, care providers, those assessing outcomes) and how | ✓ p.12 (Allocation and Blinding) |
|  | 11b | If relevant, description of the similarity of interventions | not appl. |
| Statistical methods | 12 | Methods used to address each pilot trial objective whether qualitative or quantitative | ✓ p. 24 (Analysis) |
| Results | | | |
| Participant flow (a diagram is strongly recommended) | 13a | For each group, the numbers of participants who were approached and/or assessed for eligibility, randomly assigned, received intended treatment, and were assessed for each objective | not appl. |
|  | 13b | For each group, losses and exclusions after randomisation, together with reasons | not appl. |
| Recruitment | 14a | Dates defining the periods of recruitment and follow-up | ✓ p. 19 (Data collection methods for the planned outcome measures for the future definitve trial) / p. 21 table 2 |
|  | 14b | Why the pilot trial ended or was stopped | not appl. |
| Baseline data | 15 | A table showing baseline demographic and clinical characteristics for each group | not appl. |
| Numbers analysed | 16 | For each objective, number of participants (denominator) included in each analysis. If relevant, these numbers  should be by randomised group | not appl. |
| Outcomes and estimation | 17 | For each objective, results including expressions of uncertainty (such as 95% confidence interval) for any  estimates. If relevant, these results should be by randomised group | not appl. |
| Ancillary analyses | 18 | Results of any other analyses performed that could be used to inform the future definitive trial | not appl. |
| Harms | 19 | All important harms or unintended effects in each group (for specific guidance see CONSORT for harms) | not appl. |
|  | 19a | If relevant, other important unintended consequences | not appl. |
| Discussion | | | |
| Limitations | 20 | Pilot trial limitations, addressing sources of potential bias and remaining uncertainty about feasibility | ✓ p. 26 (Strengths and Limitations) |
| Generalisability | 21 | Generalisability (applicability) of pilot trial methods and findings to future definitive trial and other studies | ✓ p. 27 (Conclusion) |
| Interpretation | 22 | Interpretation consistent with pilot trial objectives and findings, balancing potential benefits and harms, and considering other relevant evidence | not appl. |
|  | 22a | Implications for progression from pilot to future definitive trial, including any proposed amendments | not appl. |
| Other information | | |  |
| Registration | 23 | Registration number for pilot trial and name of trial registry | ✓ p. 7 (Trial registration) |
| Protocol | 24 | Where the pilot trial protocol can be accessed, if available | not appl. |
| Funding | 25 | Sources of funding and other support (such as supply of drugs), role of funders | ✓ p. 29 (Declarations) |
|  | 26 | Ethical approval or approval by research review committee, confirmed with reference number | ✓ p. 29 (Declarations) |

Citation: Eldridge SM, Chan CL, Campbell MJ, Bond CM, Hopewell S, Thabane L, et al. CONSORT 2010 statement: extension to randomised pilot and feasibility trials. BMJ. 2016;355.

*We strongly recommend reading this statement in conjunction with the CONSORT 2010, extension to randomised pilot and feasibility trials, Explanation and Elaboration for important clarifications on all the items. If relevant, we also recommend reading CONSORT extensions for cluster randomised trials, non-inferiority and equivalence trials, non-pharmacological treatments, herbal interventions, and pragmatic trials. Additional extensions are forthcoming: for those and for up to date references relevant to this checklist, see [www.consort-statement.org](http://www.consort-statement.org).
