# Supplementary material for: Implementation and evaluation of a complex intervention to improve information availability at the interface between inpatient and outpatient care in older patients with multimorbidity and polypharmacy (HYPERION-TransCare) — study protocol for a pilot and feasibility cluster-randomized controlled trial in general practice in Germany
Source: Pilot Feasibility Stud. 2023 Aug 22;9:146. doi: 10.1186/s40814-023-01375-2 (PMC10463488; doi:10.1186/s40814-023-01375-2)
Supplement: Supplementary file 3 — Additional file 3. Mixed-Methods approach of explorative process evaluation. [file 40814_2023_1375_MOESM3_ESM.docx]

Research questions and methods used in the mixed-methods approach of the explorative process evaluation (based on the framework for design and reporting of process evaluations of cluster randomized trials (Grant et al. 2013 (1)) and the Consolidated Framework of Implementation Research (CFIR, Dammschroder et al 2009 (2)).

| **Research questions** | **Planned Methods for data collection** | **Target group** | | **Data collection time point** |
| --- | --- | --- | --- | --- |
|  |  | **IG** | **CG** |  |
| (1) Expectations of intervention (CFIR) | | | | |
| What changes are expected to result from the use of the "GP practice checklist” in combination with the patient portfolio? | Qualitative semi-structured interviews of participants | HCA, GP | - | After randomization & introduction to intervention (training for GP practice) |
| Will the intervention be feasible for all participants? What, if any, are the assumed barriers? |  | HCA, GP, Patient | - |  |
|  |  |  |  |  |
|  |  |  |  |  |
| (2) Recruitment of GP practices and patients (Grant) | | | | |
| How were the GP practices recruited?  How long did the recruitment process take? (From initial enquiry until inclusion) | Analysis project documentation | GP practice | GP practice | After the intervention phase |
| What is the participation rate of GP practices?  (How) do participating vs. non-participating GP practices differ? (perhaps the GPs are different?)  Have any GP practices withdrawn their agreement to participate, if so why? | Analysis of project documentation (comparison of practice structure between participating/non-participating GP practices in the SaxoForN database) | GP practice | GP practice | After the intervention phase |

| What factors motivate/hinder GP practices to/from study participation? | Short survey of  (non-)participating GP practices | GP practice | GP practice | Short survey with non-participating practices |
| --- | --- | --- | --- | --- |
|  |  |  |  | Short interview with participating practices (after intervention phase) |
| How were patients recruited? Did recruitment depend on specific characteristics?  What is the participation rate? Overall and per GP practice.  (How) do participating patients differ from non-participating patients?  In case of dropouts: Retention rate and for what reason(s)? | Analysis of GP practice recruitment protocols, qualitative semi-structured interviews of participants | GP practices, Patients | GP practices, Patients | After the intervention phase |
| (3) Implementation of the study design at practice level (Grant) | | | | |
| Was the study conducted as planned?    Patient inclusion as planned? Difficulties if any (inclusion criteria)?  Recruitment process as planned?  Cluster randomization as planned?  Outcome measurements as planned (measurable in individual sub-groups: IG, CG, with/without hospital stays).  Time schedule adhered to consistently throughout all processes? | Analysis of project documentation in GP practices and study teams | Study teams | Study teams | Summative evaluation after intervention phase |

| (4) Intervention implementation at practice level (Grant & CFIR) | | | | |
| --- | --- | --- | --- | --- |
| What did the practices think of the implementation of the intervention and training? How useful were the trainings, is there potential for optimization? What concerns were there? | Quantitative evaluation of the training | GP practice, Patient | GP practice | After completion of training and after the information on the intervention had been distributed |
|  |  |  |  |  |
|  |  |  |  |  |
| How was the intervention implemented after training of the GP practices?  "GP practice checklist”;  patient portfolio;  if necessary, coordinate discussion in GP practice | Qualitative analysis of project documentation, short surveys | GP practice | - | Summative evaluation after intervention phase |
|  |  | Patient, GP practice | - |  |
|  |  | GP practice, Patient | - |  |
| (5) Intervention (Grant & CFIR) | | | | |
| How were the "GP practice checklists” used in the GP practices? Changes over time?  How often were patient portfolios reviewed by the HCA? Changes over time?  What data in the patient portfolio were considered to be important.  Did structural factors influence usage? | Analysis of project documentation, Qualitative semi-structured interviews of participants | GP practice, Patient | - | After the intervention phase |

| How were patient portfolios used by patients? Were patient portfolios filled in, maintained and regularly taken to doctor's appointments?  Did their use change over time (maintenance)?  What health/personal characteristics can explain differences in use? | Check the portfolios for completeness and information flow (contact details, comparison of FIMA for patient and GP practice, as well as patient portfolio score/FIMA), Qualitative semi-structured interviews of participants | Patient, GP practice | - | After the intervention phase |
| --- | --- | --- | --- | --- |
| If applicable, was the patient portfolio taken to the hospital and used by staff? How was the portfolio evaluated? | Feedback cards with specific questions included in patients’ portfolios | Hospital staff | - | During intervention |
| What role did the HCA/GP practice play in maintaining the patient’s portfolio?  Should any changes be made to the intervention´s components? | Qualitative semi-structured interviews of participants | Patient, HCA | - | After intervention phase |
|  |  |  |  |  |
| (6) Feedback on the intervention/intervention implementation (CFIR) | | | | |
| From the patients' point of view, is it beneficial to have more information about themselves?  What content of the portfolio did patients consider important and what content did they fail to keep up to date?  Did they have any concerns? Is the intervention lacking important content and if so, what. | Qualitative semi-structured interviews of | Patient | Patient | After intervention phase |
|  |  |  |  |  |
|  |  |  |  |  |

| Can the "GP practice checklist” and the patient portfolio provide information that is helpful in case of hospitalization?  Ideas on optimization? | Qualitative semi-structured interviews | GP practice, Patient | - | After intervention phase |
| --- | --- | --- | --- | --- |
| What chances and risks are seen in the everyday use of the intervention by GP practices and patients?  What supporting structures are necessary? | Qualitative semi-structured interviews | GP practice, Patient | - | After intervention phase |
| Did study participation motivate patients to adopt other health-promoting behaviors (efforts to prevent falls, improved adherence, etc.)? | Interview (written or oral) | GP practice, Patient | GP practice, Patient | After intervention phase |
|  |  |  |  |  |
|  | | | | |

Literature Cited

1. Grant A, Treweek S, Dreischulte T, Foy R, Guthrie B. Process evaluations for cluster-randomised trials of complex interventions: a proposed framework for design and reporting. Trials 2013; 14:15. Available from: URL: https://pubmed.ncbi.nlm.nih.gov/23311722/.

2. Damschroder LJ, Aron DC, Keith RE, Kirsh SR, Alexander JA, Lowery JC. Fostering implementation of health services research findings into practice: a consolidated framework for advancing implementation science. Implementation science : IS 2009; 4:50. Available from: URL: https://pubmed.ncbi.nlm.nih.gov/19664226/.
